# Near-Infrared Spectroscopy Intravascular Ultrasound Imaging: State of the Art

**DOI:** 10.3389/fcvm.2020.00107

**Published:** 2020-06-30

**Authors:** Kayode O. Kuku, Manavotam Singh, Yuichi Ozaki, Kazuhiro Dan, Chava Chezar-Azerrad, Ron Waksman, Hector M. Garcia-Garcia

**Affiliations:** ^1^MedStar Cardiovascular Research Network, MedStar Washington Hospital Center, MedStar Health Research Institute, Washington, DC, United States; ^2^Section of Interventional Cardiology MedStar Washington Hospital Center, MedStar Heart and Vascular Institute, Washington, DC, United States; ^3^Georgetown University Department of Medicine, Washington, DC, United States

**Keywords:** near-infrared spectroscopy (NIRS), lipid-rich plaque, plaque-characterization, intravascular imaging, intravascular ultrasound (IVUS), coronary artery disease, coronary plaque

## Abstract

Acute coronary syndromes (ACS) secondary to coronary vessel plaques represent a major cause of cardiovascular morbidity and mortality worldwide. Advancements in imaging technology over the last 3 decades have continuously enabled the study of coronary plaques via invasive imaging methods like intravascular ultrasound (IVUS) and optical coherence tomography (OCT). The introduction of near-infrared spectroscopy (NIRS) as a modality that could detect the lipid (cholesterol) content of atherosclerotic plaques in the early nineties, opened the potential of studying “vulnerable” or rupture-prone, lipid-rich coronary plaques in ACS patients. Most recently, the ability of NIRS-IVUS to identify patients at risk of future adverse events was shown in a prospective multicenter trial, the Lipid-Rich-plaque Study. Intracoronary NIRS-IVUS imaging offers a unique method of coronary lipid-plaque characterization and could become a valuable clinical diagnostic and treatment monitoring tool.

## Introduction

Coronary artery disease (CAD) has continued to be a major cause of morbidity and mortality worldwide, despite recent advances in medical and interventional therapies (1). The pathogenesis of acute coronary events involves the development of an early core with the accumulation of lipid-rich free cholesterol which then progresses to the formation of a fibroatheroma (2). Coronary angiography has been a crucial tool for detecting the gross presence of disease of coronary lesions, it however underestimates the magnitude of atherosclerosis in non-culprit arteries particularly in the early stages of disease process and provides no information regarding the composition of the plaque responsible for the lesion. Intracoronary imaging including intravascular ultrasound (IVUS) and near infrared spectroscopy (NIRS) have been studied to determine the plaque burden (PB) and plaque composition, respectively.

Intravascular Ultrasound provides 2-dimensional cross-sections of arterial vessels which enables the visualization and characterization of not just lumen and stent struts, but the vessel wall dimensions and the presence of plaques within it. In spite of the usefulness of IVUS in the study of plaques and vessel remodeling, it remains limited in the visualization and quantification of certain plaque characteristics mainly due to the inherent properties of sound waves (3). NIRS imaging offers the ability to penetrate blood and tissue to detect lipid core containing coronary plaques. The technology is based on the property of different organic molecules to scatter and absorb light at different intensities and wavelengths (4, 5). The integration of NIRS and IVUS systems has provided a hybrid imaging modality which combines the penetration and high resolution of IVUS with the lipid core quantification and characterization of NIRS which has been demonstrated to correlate with the lipid detection (6, 7).

We will review the present and potential clinical and research utility of NIRS-IVUS imaging in the study of coronary lipid plaques in the context of CAD.

## Near Infrared Spectroscopy Technique

Near-infrared diffuse reflectance spectroscopy is a technique that relies on the property of substances to absorb and scatter NIR light (wavelengths from 800 to 2,500 nm) at different intensities as a function of wavelengths (8–10). NIRS as a technique has been used in various science fields, including chemistry and pharmaceuticals, for the determination of the chemical composition of substances.

Prior to the development of NIRS, various spectroscopy techniques (nuclear magnetic resonance spectroscopy, Raman spectroscopy, and fluorescence spectroscopy) had been studied for possible intravascular applications toward the study of atherosclerotic plaques. The Raman near-infrared spectroscopy (NIRS) which was widely used several disciplines was based on the inelastic scattering of photons following collision with molecules and while it was suggested to have the potential to identify vulnerable plaques, was limited by signal-to-noise problems (11–14). The NIRS technology was first used in 1993 by Cassis and Lodder in animal experiments in which they sought the characterization of low-density lipoprotein cholesterol accumulation in the aortas of hypercholesterolaemic rabbits. This was followed by the *in-vivo* use of diffuse reflectance NIRS in imaging the lipid content in human carotid plaques exposed during surgery (15, 16)

### Validation of NIRS

In the early days of NIRS, 2 pivotal studies were carried out to validate its accuracy for the detection of lipid core plaques (LCPs) in human vessels. The first study by Gardner et al. made use of 84 human heart specimens- 33 hearts were used to develop NIRS algorithms and produce predefined endpoints while the remaining 51 hearts were used for prospective validation of algorithm, in a double-blinded study design, to evaluate the accuracy of NIRS in detecting LCPs. In order to have a quantitative target for constructing the algorithm and validating the findings, an LCP of interest was defined as a “fibroatheroma” (FA) with a lipid core >60° in circumferential extent, >200 μm thickness, and with a fibrous cap of mean thickness <450 μm.” The primary analysis which was done by comparing NIRS information presented on block chemogram readings vs. the classified histologic findings showed a “receiver operating characteristic (ROC) area under the curve (AUC) of 0.80 (95 % CI: 0.76–0.85),” confirming the ability of the NIRS system to accurately identify the LCPs (4).

Secondly, the Spectroscopic Assessment of Coronary Lipid (SPECTACL) study which was the first catheter-based technique to use NIRS in humans for percutaneous application was performed to validate the applicability of the autopsy-based LCP detection algorithm in patients. The study, in addition to showing that the NIRS imaging catheter had a similar safety profile to that of IVUS, demonstrated that the spectra obtained from imaging the epicardial vessels of living patients were similar to those from previously validated spectra from autopsy specimens, thereby supporting the use of NIRS for detection of LCPs in human patients (9).

Earlier, several *ex-vivo* studies had examined the ability of NIRS to identify histological features of lipid-rich atherosclerotic plaques in human blood vessels obtained at autopsy. These studies reported >90% sensitivity and specificity for the identification of characteristic features suggesting lipid-rich plaques including the rupture-prone thin-cap fibroatheromas (TCFAs) seen in ACS patients. More recent studies have corroborated these findings as well as pointing to the additive value of NIRS to IVUS-derived PB in detecting vulnerable plaques (17–21).

Intra- and Inter-catheter reproducibility of the NIRS catheter has also been validated in a number of independent studies (22, 23).

### Principles of Near Infrared Spectroscopy-Intravascular Ultrasound (NIRS-IVUS) Catheter

A little over a decade ago, a single modality NIRS system was originally developed for the invasive detection of lipid core plaques (LipiScan™, Infraredx Inc., Bedford, MA, USA). In later years, a dual modality system which combined IVUS with NIRS was developed to provide in a single catheter information on both vessel structure and plaque composition in a single acquisition. The NIRS-IVUS systems have continued to evolve and now exist in the form of a dual frequency, dual modality system. (TVC Imaging System™ and Makoto Intravascular Imaging System™, Infraredx Inc.) Table 1.

**Table 1:** Evolution of NIRS/NIRS-IVUS imaging-based systems.

**Trade name**	**Model number**	**Year introduced (US)**	**Features/design specifications**	**Advantages/improvements/revisions**	**Additional comments**
LipiScan	NIRS-MC5	2008	NIRS (8,000 NIRS spectra/100 mm)	First FDA cleared NIRS imaging System with two-dimensional map of LCP	
LipiScan IVUS	TVC-MC7	2010	NIRS (8,000 NIRS spectra/100 mm); 40 MHz, Grayscale IVUS	First FDA cleared dual imaging system which combined co-registered, grayscale, 40 MHz IVUS with proprietary NIR LCP detection technology to identify LCPs, degree of stenosis, reference vessel diameter, and plaque burden	
TVC Imaging System	TVC-MC8	2012	NIRS (32,000 NIRS spectra/100 mm); 40 MHz, Grayscale IVUS	First multimodality imaging to combine IVUS and NIRS. Improved IVUS Image quality and resolution with hydrophilic coating on catheter and added manual IVUS imaging features.	1. Lower frequency increases depth-of-field while the higher frequency improves clarity. 2. Improved resolution enhancing visualization of vessel details
Advanced TVC Imaging System	TVC-MC8x	2014	NIRS (32,000 NIRS spectra/100 mm); 35–65 MHz, Grayscale IVUS	High definition “HD” IVUS Image Quality with dual-modality frequency (up to 65 MHz) capabilities. Dual-layer hydrophilic coating. 40-micron axial resolution	
TVC Imaging System	TVC-MC9	2015	NIRS (32,000 NIRS spectra/100 mm); 35–65 MHz, Grayscale IVUS	Enhanced user interface and IVUS image with 20-micron axial resolution. Extended bandwidth rotational IVUS catheter.	
Makoto Imaging System	TVC-MC10	2019	NIRS (1,300 NIRS spectra/mm); 35–65 MHz IVUS	User Interface Enhancements. Multiple (0.5, 1.0, 2.0 mm/s) Pullback Speeds. 0.0, 2.0, 10.0mm/s Manual IVUS tip movement speed	

*NIRS, Near-Infrared spectroscopy; FDA, Food Drug and Administration; TVC, True Vessel Catheterization; LCP, Lipid Core Plaque; IVUS, Intravascular Ultrasound; MHz, Megahertz (IVUS Transducer frequency)*.

**Figure 1:**
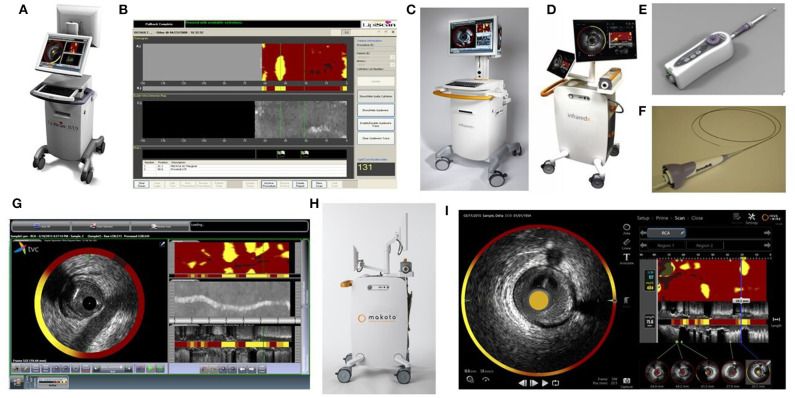
NIRS Prototype figures **(A)** LipiScan-NIRS-MC5 **(B)** LipiScan-NIRS-MC5 Display **(C)** 2012 TVC Imaging System-MC8 **(D)** 2015 TVC Imaging System-MC9 **(E)** NIRS Pullback device **(F)** NIRS-IVUS catheter **(G)** 2012 TVC Display **(H)** Makoto Imaging System- MC10 **(I)** 2018 Makoto Display (All images in this Figure are licensed content by InfraRedX Inc. and shall not be reproduced).

The NIRS-IVUS system comprises a scanning NIR laser, a pullback and rotation unit, and a traditional IVUS-sized catheter. The 3.2F rapid exchange catheter has an entry profile of 2.4F and a shaft profile of 3.6F and is compatible with 6F guiding catheters. The NIRS-IVUS can be inserted over a 0.014-inch guide wire while its passage through the lesion is facilitated by the hydrophilic coating present on the flexible distal 50 cm end. The IVUS images are acquired during an automated pullback with simultaneous co-registered NIRS measurements. In the latest generation, the Makoto system, the catheter's imaging core pulls back at speeds of 0.5, 1.0, or 2.0 mm/s and rotates at 1800 rpm with a maximum imaging length of 15 cm, acquiring up to ~130,000 NIRS per 100 mm (4, 9, 24) (Figure 1).

**Figure 2:**
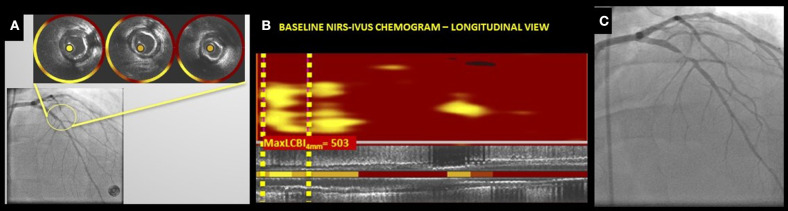
**(A–C)** NIRS-IVUS chemograms in non-culprit lesions showing LCBI in non-stenotic angiographic segments; **(A)** Baseline Angiogram cine showing left anterior descending (LAD) segment with IVUS cross-sections and NIRS-IVUS rings indicating the presence of lipid plaque, **(B)** Baseline NIRS-IVUS chemogram (longitudinal view) showing the calculated maximum 4 mm LCBI (max4mmLCBI) in a 4 mm segment of the LAD segment shown in **(A)**. **(C)** Follow up angiogram of the same vessel segment showing a severely stenotic segment in the proximal LAD. (Courtesy of the MedStar Health Research Institute, MedStar Cardiovascular Research Network, Invasive Imaging Core Laboratory).

NIRS lipid core data are automatically displayed on a “chemogram” which displays the probability of the presence of a lipid rich plaque with the millimeters of pull-back on the x-axis and the circumferential position on the y-axis. Areas containing lipid core are displayed as yellow and those without any significant lipid core as red. A quantitative image metric is automatically reported as a numerical lipid-core burden index (LCBI), which represents the fraction of the chemogram yellow pixels whose probability of lipid exceeds 0.6, per 1,000. The LCBI provides a quantitative metric of the lipid core plaque present in a scanned vessel and can be computed over the entire length of a vessel scan, segments of scans, or defined width windows within segments (such as the 4 mm sliding window with the maximum LCBI, maxLCBI4mm) (9) (Figure 2).

## Applications of NIRS-IVUS

### Lipid Rich Plaque Characterization

Prior autopsy studies have shown that lesions with a thin fibrous cap(<65 mn of cap thickness) overlying a large necrotic core are most frequently prone to rupture (4, 25, 26). These “vulnerable” plaques have therefore become a target for identification by novel intracoronary imaging modalities. Several studies in ACS patients have since shown the presence of LCPs in the culprit arterial segments to be major precursors of the disease and the possible association of the lipid core burden with adverse cardiovascular outcomes in CAD patients overtime (27–34) (Table 2).

**Table 2:** Overview of key studies evaluating the utility and efficacy of NIRS-IVUS imaging in CAD.

**Study**	**Year**	**Study aim**	**Study design/**	**Sample size reported**	**Follow-Up duration**	**NIRS related endpoint**	**Key merits**	**Limitations**	**Main conclusion**
The COLOR Trial (35)	2011	To determine whether intracoronary NIRS can identify plaques that are likely to cause periprocedural MI in patients undergoing elective PCI	Prospective observational	62		The rate of periprocedural MI in the groups with and without a large LCP in the treatment zone as assessed by NIRS and expressed as maxLCBI4 mm	1. Showed the relationship between the risk of periprocedural MI and NIRS-detected LCPs; 2. Included a comparison group of patients without large LCBI	1. Small sample size; 2. Selection bias due to the availability of post-PCI biomarkers	NIRS imaging provides a rapid and automated means of LCP identification can be used to identify large, stenotic, coronary LCPs, which in the study were found to be associated with a 50% risk of periprocedural MI when dilated during PCI
The YELLOW Trial (36)	2013	To evaluate the effect of short-term statin therapy on intracoronary plaque using FFR and NIRS-IVUS system in patients with multivessel CAD undergoing PCI and with at least 1 severely obstructive (FFR ≤ 0.8) non-culprit	Randomized Clinical Trial	87	7 weeks	Change in lipid-core burden index at the LCBI4 mm max segment	1. Evaluated the effect of therapeutics on NIRS- quantified lipid content; 2. NIRS imaging performed at baseline and follow-up; 3. Included correlation of physiology (FFR) against NIRS-derived LCBI; 4. Randomized study design	1. Small sample size; 2. Short follow-up duration; 3. IVUS and NIRS were performed using separate catheters 4. Differences in baseline LCBIs between the two study groups	Significant reduction in maxLCBI4mm in the intensive statin group vs the standard of care group
ATHEROREMO-NIRS Oemrawsingh et al. (37)	2014	To determine the long-term prognostic value of intracoronary NIRS as assessed in a non-culprit vessel in patients with CAD	Prospective observational	203	12 months	Composite of all-cause mortality, non-fatal ACS, stroke, and unplanned coronary revascularization exclusive of culprit lesion events	1. Clinical endpoints; 2. Results strongly suggested the prognostic value of NIRS imaging in non-stenotic, non-culprit segments	1. Small sample size; 2. A single non-culprit coronary artery was imaged per patient	“CAD patients with an LCBI equal to or above the median of 43.0, as assessed by NIRS in a non-culprit coronary artery, had a 4-fold risk of adverse cardiovascular events at 1-year follow-up These findings relate to the risk throughout the entire coronary tree and not necessarily at the imaged segment or a lesion-specific risk”
The CANARY Trial (38)	2015	To determine whether pre-PCI plaque characterization using NIRS is capable of identifying lesions at risk of periprocedural myonecrosis	Prospective Randomized Pilot Trial	85		Incidence of periprocedural MI, defined as troponin or creatine kinase-myocardial band increase to 3 or more times the upper limit of normal within 72 h	1. Randomized design; 2. Correlation of NIRS measures vs. MI enzyme parameters; 3. Confirmed a relationship between NIRS-identified LRPs and periprocedural myonecrosis	1. Small study size; 2. Not all clinically relevant MIs.	Pre-interventional intravascular imaging with a combined NIRS-IVUS catheter is able to identify lesions at increased risk of periprocedural myonecrosis after stent implantation Furthermore, the use of a
IBIS-3 (39, 40)	2016	To evaluate the effect of high intensity statin therapy on compositional coronary plaque changes using RF-IVUS and NIRS in non-culprit segments	Prospective observational	103	6 months-12 months	The effect of high intensity-rosuvastatin on LCP within non-stenotic NCLs	1. Evaluated the effect of lipid lowering therapeutics on NIRS-quantified LCBI in non-stenotic segments; 2. Included study of the stability of the plaque necrotic core; 3. NIRS imaging at baseline and follow-up	1. Lack of a randomized design (no comparison group); 2. Events incidence not sufficient to assess MACE	High dose rosuvastatin therapy resulted in non-significant change in Necrotic Core (NC) and a neutral effect was observed in LCBI
The YELLOW II Trial (41)	2017	Assess changes in plaque morphology using intravascular imaging, and evaluate cholesterol eflux capacity in stable multivessel CAD patients receiving high-dose statin therapy	Prospective observational	85	8–12 weeks	To examine lipid content changes in obstructive NCLs, measured by NIRS, and plaque morphology, assessed by OCT; and compare changes in lipid content and plaque morphology with changes in LDL, HDL, apo A-I, and macrophage functionality	1. Study design included genetic, clinical and plaque parameters 2. Multimodality imaging- Included OCT imaging in assessing plaque	1. Lack of a randomized design/ comparison group 2. Short follow-up duration	There was no significant change observed in plaque lipid content quantified using NIRS. But a significant increase in FCT of obstructive NCLs and enhancement of CEC in patients with stable coronary artery disease
LRP Study (42, 43)	2019	To establish the relationship between LRPs detected by NIRS-IVUS at the non-culprit sites and subsequent coronary events from new culprit lesions	Prospective cohort	1,271	24 months	Non-Index Culprit Lesion related Major Adverse Cardiac Events (NC-MACE) in patients and plaque in association with maxLCBI4 mm	1. Large multicenter study design with clinical endpoints; 2. Included plaque and patient level endpoints; 3. Largest intracoronary imaging study to identify patients and vessel segments at risk of future clinical events	1. Follow-up duration not extending beyond 2 years; 2. Only a few patients had all 3 major vessels scanned (average of 2.1 vessels scanned/patient)	Coronary segments at higher risk of subsequent NC-MACE were associated with higher analyzable maxLCBI_4mm_ > 400

**The PROSPECT** (Providing Regional Observations to Study Predictors of Events in the Coronary Tree) Trial, a landmark study which included 697 acute coronary syndrome (ACS) patients was a natural history study that sought to provide *in vivo* evidence of the hypothesis that the histopathological characteristic of plaques and not the degree of stenosis on angiography was responsible for the development of ACS. The study investigated the non-culprit coronary lesions in patients with ACS who underwent three-vessel gray scale and VH-IVUS imaging after successful PCI of culprit lesion demonstrated that “independent predictors of non-culprit-related events were the presence of VH-IVUS thin-cap fibroatheroma (hazard ratio 3.35; 95% confidence intervals 1.77–6.36), a PB ≥70% (hazard ratio 5.03; 95% confidence intervals 2.51–10.11), and a minimum lumen area ≤ 4.0 mm^2^ (hazard ratio 3.21; 95% confidence intervals 1.61–6.42)” (44).

Following the results from the PROSPECT Study, The **COLOR** registry prospectively observed a positive association between plaque morphology evaluated by NIRS and the degree of coronary artery stenosis. It demonstrated that increasing degree of stenosis seen by angiography was associated with more vulnerable plaque morphology as assessed by NIRS-IVUS system. Also, one of the first prospective human studies which evaluated high LCP by NIRS vs. cardiovascular events- The ATHEROREMO-NIRS (The European Collaborative Project on Inflammation and Vascular Wall Remodeling in Atherosclerosis—Near-Infrared Spectroscopy) trial, a sub-study of the ATHEROREMO-IVUS study of nearly 600 patients (to evaluate some of the limitations of VH IVUS identified after the PROSPECT and VIVA studies) produced promising results regarding the possibility of the technology. The relationship of LCBI value with the primary endpoint composite was found to be similar in both stable angina and ACS patients. In a more recent study, Matsumura et al. employed NIRS-IVUS in examining the features of coronary lesions with intraplaque hemorrhage (a key culprit in coronary lesion progression) in a histopathological validation study and demonstrated the presence of more FAs, greater IVUS PB and NIRS lipid core burden present in intraplaque hemorrhage segments compared to segments without intraplaque hemorrhage (35, 37, 45–47) Table 2.

In the most recent prospective multicenter natural history intracoronary imaging study—**The LRP (Lipid Rich Plaque)** identified patients and coronary segments at risk of future major adverse coronary events using NIRS-IVUS system. Patients with known or suspected CAD undergoing cardiac catherization with possible PCI were examined via NIRS-IVUS imaging in non-culprit arteries when possible. The study showed that 9% of the patients had subsequent non-culprit-major adverse cardiac events (NC-MACE) and out of these patients, higher event rate was associated with analyzable maxLCBI_4mm_ > 400 (HR 3.39; 95% confidence interval 1.85–6.20), pointing to the diagnostic value of the NIRS-IVUS (42, 43). Following these results, the FDA granted a label claim for NIRS detection and identification of patients at increased risk of major adverse cardiac events (MACE) Table 2.

### Clinical Applications of Intracoronary Near-Infrared Spectroscopy

In general, the early evidence from NIRS-IVUS use points to multiple potential clinical applications of this imaging system. The identification and localization of vulnerable atherosclerotic plaques offers a clinical tool that could help assess precise lesion lengths including segments with a high lipid core burden toward optimal stenting (48). Secondly, NIRS-IVUS offers information on the necrotic core of atherosclerotic plaques and can predict the embolization of highly thrombogenic lipid depositions. The release of these elements into the blood stream which results in distal embolization is a known culprit in peri-procedural myonecrosis (35, 38, 49–51). Similarly, the utility of NIRS-IVUS in optimizing carotid artery stenting toward preventing periprocedural stroke is a subject of ongoing research (52, 53).

Furthermore, NIRS-IVUS has been employed in the study of plaque morphology and composition in the peripheral arteries-superficial femoral arteries in the setting of severe stenoses and symptomatic peripheral artery disease (PAD) (54, 55).

Several other studies (37, 43, 56, 57) which show the association of either max4mmLCBI or high LCBI (in the case of Danek et al. and Oemrawsingh et al.) in non-target/non-culprit vessel segment with increased incidence of MACCE and the risk of future coronary events subsequent to the presence of vulnerable plaques in non-culprit segments has given credence to retrospective autopsy studies regarding the assessment of plaque vulnerability. The ability of NIRS-IVUS to assess the possibility of future coronary events based on the LCBI values in non-culprit segments offers a unique diagnostic utility and a risk-stratification tool. The predictive role of NIRS-IVUS shown in these studies is being further studied in ongoing studies and raises the question of the need for treating non-culprit vulnerable plaques in vulnerable patients.

With the evolution of pharmacological therapies in addition to the performance of the present lipid-lowering therapies, NIRS-IVUS offers a tool for monitoring the effects of medications such as statins and PCSK9-inhibitors on the coronary vasculature lipid burden. Already, studies have investigated the effect of lipid-lowering medications on reducing necrotic-core containing plaques. The YELLOW (Reduction in Yellow Plaque by Aggressive Lipid-Lowering therapy) trial was a randomized clinical trial (rosuvastatin 40 mg daily vs. the standard-of-care lipid-lowering therapy) which recruited patients with multi-vessel CAD (including at least 1 severely obstructive [FFR ≤ 0.8] non-culprit) undergoing revascularization by PCI. The non-target lesions in both groups were evaluated at baseline and following 7 weeks of therapy with fractional flow reserve (FFR), and NIRS-IVUS, comparison between baseline and follow up results for NTLs demonstrated that reduction in LCBI4mm max was significantly higher in intensive statin group as compared to standard group (36) In a follow up study -the YELLOW II, the effects of high-dose statin therapy on changes in plaque morphology (evaluated by OCT) and plaque lipid content by NIRS were assessed in obstructive non-culprit lesions (NCLs) in addition to a comprehensive assessment of cholesterol efflux capacity (CEC) and peripheral blood mononuclear cell (PBMC) transcriptomics. The mean baseline NIRS-derived maxLCBI4mm was over 400 (416.6 ± 172.9) and the study reported no significant change in plaque lipid content by NIRS albeit a significant change in fibrous cap thickness after about 12 weeks of statin therapy. (41). Almost similarly, the IBIS-3 (Integrated Biomarker and Imaging Study 3) trial demonstrated that “high dose rosuvastatin therapy had a neutral effect on LCP (assessed by NIRS LCBI) in non-stenotic NCLs in the coronary vessels after 6- and 12-month follow-up intervals despite a relatively lower mean baseline maxLCBI4mm (201.9 ± 1623.8), in the cohort of 103 patients”(39, 40). While differences in LCBI cut-offs of the various studies could be a possible reason for the discrepancy in findings, the mean baseline maxLCBI4mm in the YELLOW I and YELLOW II trials were similar, even though the baseline LCBI was significantly higher in patients randomly allocated to the intensive vs. standard therapy group in the YELLOW I and there was no comparative standard therapy group in the YELLOW II. There is also the question of short term LRP/LCBI regression occurring mostly in plaques with a large PB and large LRPs reported in a YELLOW trial sub-study evaluating the relationship between LRPs, plaque morphology and lesion progression or regression. These findings point to the need for further exploration of the effect of statins as well as novel lipid-lowering therapies on NIRS-derived LCPs and LCBI in conjunction with plaque morphology analysis via other intracoronary modalities (58).

## Future Directions

The findings of Waksman et al. in the recent LRP study (43) strongly suggest the viability of NIRS-IVUS as a diagnostic and risk-stratifying modality and opens the door to further trials to evaluate therapeutic strategies against high lipid core burden and/or vulnerable plaques/patients in addition to studies which could explore the value of LCBI as a surrogate marker for the checking the effectiveness of new therapeutic interventions.

Notably, the effects of newer LDL-lowering medications are currently being studied in ongoing trials. The PACMAN AMI (Vascular Effects of Alirocumab in Acute MI-Patients) trial (ClinicalTrials.gov Identifier: NCT03067844) is examining the effects of PCSK9-inhibiting monoclonal antibody, alirocumab on coronary atherosclerosis in acute MI patients via NIRS, IVUS and OCT. Also, the FITTER (Functional Improvement of Coronary Artery Narrowing by Cholesterol Reduction With a PCSK9 Antibody) Trial (ClinicalTrials.gov Identifier: NCT04141579), will be enrolling patients to investigate the impact of evolocumab plus statins on FFR of non-infarct related arteries (non-IRAs) in multivessel disease patients and correlating baseline NIRS-derived lipid core burden with changes in FFR in the non-IRAs.

In a study design which integrates a natural history study and a randomized trial, the PROSPECT II and PROSPECT ABSORB, ClinicalTrials.gov Identifier: NCT02171065), patients with high risk plaque were randomized to receive Absorb BVS alongside the standard of care, optimal medical therapy (OMT) or vs. OMT alone and changes in the plaque will be evaluated by IVUS and NIRS at follow up. Lastly, The Preventive Coronary Intervention on Stenosis With Functionally Insignificant Vulnerable Plaque (PREVENT) study, is presently recruiting patients to determine the effect of preventive PCI on functionally insignificant coronary lesions with vulnerable plaque characteristics using NIRS, IVUS, OCT and virtual histology-IVUS modalities (ClinicalTrials.gov Identifier: NCT02316886).

While the value of NIRS-IVUS and other intracoronary imaging techniques are being shown in various imaging trials, there are still limitations in assessing plaque characteristics which could explain some of the conflicting study results. Multimodality imaging may be critical in overcoming these limitations, thus emphasizing the need for co-registration of NIRS-IVUS with other imaging including angiography and as well as OCT or in the form of hybrid imaging catheters (59). Finally, the ability of NIRS-IVUS to predict the location of histologically-confirmed TCFA and the possibility of NIRS-guided cap thickness detection, and collagen content analysis which are already being explored in *ex-vivo* studies could represent a huge breakthrough in vulnerable plaque studies in the near future.

## Limitations

One of the main limitations of NIRS-IVUS is the invasiveness of the technique which precludes its use in primary prevention in symptomatic patients with subclinical disease. Presently, NIRS imaging does not have the capability to detect the depth of the lipid core or vulnerable plaque features such as the thinness of the fibrous cap.

## Conclusion

NIRS in conjunction with IVUS is certainly a diagnostically useful tool for the detection of vulnerable plaques and can help identify patients at risk of future coronary events. While there is a wide spectrum of clinical applications for this technology, its viability for demonstrating the effects of present and forthcoming lipid-lowering therapies could significantly influence clinical perspectives and practice in the years to come.

## Author Contributions

All authors made a substantial contributions to the conception or design of this review paper, including the drafting and revisions. All authors have granted their approval for all aspects the manuscript and its submission.

## Conflict of Interest

RW has served on advisory boards for Amgen, Boston Scientific, Cardioset, Cardiovascular Systems, Medtronic, Philips Volcano, and Pi-Cardia; has served as a consultant for Amgen, Biosensors, Biotronik, Boston Scientific, Cardioset, Cardiovascular Systems, Medtronic, Philips Volcano, and Pi-Cardia; and has received grant support from AstraZeneca, Biotronik, Boston Scientific, and Chiesi; and has participated at a speakers bureau for AstraZeneca and Chiesi; is an investor in MedAlliance. The remaining authors declare that the research was conducted in the absence of any commercial or financial relationships that could be construed as a potential conflict of interest.

## Abbreviations

ACS: Acute Coronary Syndrome
IVUS: Intravascular ultrasound
OCT: Optical Coherence Tomography
NIRS: Near infrared spectroscopy
PB: Plaque Burden CAD: Coronary Artery Disease
PCI: Percutaneous Coronary Intervention
LCP: lipid core plaques
LRP: Lipid rich plaque
FA: Fibroatheroma
ROC: Receiver Operating Characteristic
AUC, Area Under the Curve: TVC: True Vessel Characterization
LCBI: Lipid core burden index
NC-MACE: Non culprit major cardiovascular events
MACE: Major adverse cardiac events
FDA: Food and Drug Administration
TCFA: thin-capped fibroatheromas
PAD: Peripheral arterial disease
MACCE: Major Adverse cardiovascular and cerebrovascular events
FFR: Fractional Flow Reserve
RF-IVUS: Radiofrequency IVUS
BVS: Bioresorbable vascular scaffold.

## References

[1] Benjamin EJ Muntner P Alonso A Bittencourt MS Callaway CW Carson AP. Heart disease and stroke statistics-2019 update: a report from the American Heart Association. Circulation. (2019) 139:e56–528. 10.1161/CIR.0000000000000659 30700139

[2] Guyton JR Klemp KF. Development of the lipid-rich core in human atherosclerosis. Arterioscler Thromb Vasc Biol. (1996) 16:4–11. 10.1161/01.ATV.16.1.4 8548424

[3] Garcia-Garcia HM Costa MA Serruys PW Imaging of coronary atherosclerosis: intravascular ultrasound. Eur Heart J. (2010) 31:2456–69. 10.1093/eurheartj/ehq280 20823109

[4] Gardner CM Tan H Hull EL Lisauskas JB Sum ST Meese TM. Detection of lipid core coronary plaques in autopsy specimens with a novel catheter-based near-infrared spectroscopy system. JACC Cardiovasc Imaging. (2008) 1:638–48. 10.1016/j.jcmg.2008.06.001 19356494

[5] Friebel M Helfmann J Netz U Meinke M. Influence of oxygen saturation on the optical scattering properties of human red blood cells in the spectral range 250 to 2,000 nm. J Biomed Opt. (2009) 14:034001. 10.1117/1.3127200 19566295

[6] Roleder T Wojakowski W Intravascular ultrasound, optical coherence tomography and near infrared spectroscopy. Cor et Vasa. (2015) 57:e439–45. 10.1016/j.crvasa.2015.10.004

[7] Yonetsu T Suh W Abtahian F Kato K Vergallo R Kim SJ. Comparison of near-infrared spectroscopy and optical coherence tomography for detection of lipid. Catheter Cardiovasc Interv. (2014) 84:710–7. 10.1002/ccd.25084 23785015

[8] Cao Q Zhegalova NG Wang ST Akers WJ Berezin MY. Multispectral imaging in the extended near-infrared window based on endogenous chromophores. J Biomed Opt. (2013) 18:101318. 10.1117/1.JBO.18.10.101318 23933967 PMC3739874

[9] Waxman S Dixon SR L'Allier P Moses JW Petersen JL Cutlip D. *In vivo* validation of a catheter-based near-infrared spectroscopy system for detection of lipid core coronary plaques: initial results of the SPECTACL study. JACC Cardiovasc Imaging. (2009) 2:858–68. 10.1016/j.jcmg.2009.05.001 19608137

[10] Caplan JD Waxman S Nesto RW Muller JE. Near-infrared spectroscopy for the detection of vulnerable coronary artery plaques. J Am Coll Cardiol. (2006) 47:C92–6. 10.1016/j.jacc.2005.12.045 16631516

[11] Schaeberle MD Kalasinsky VF Luke JL Lewis EN Levin IW Treado PJ. Raman chemical imaging: histopathology of inclusions in human breast tissue. Anal Chem. (1996) 68:1829–33. 10.1021/ac951245a 8686910

[12] Zhang Y Hong H Cai W Imaging with raman spectroscopy. Curr Pharm Biotechnol. (2010) 11:654–61. 10.2174/138920110792246483 20497112 PMC2917525

[13] Butler HJ Ashton L Bird B Cinque G Curtis K Dorney J. Using raman spectroscopy to characterize biological materials. Nat Protoc. (2016) 11:664–87. 10.1038/nprot.2016.036 26963630

[14] Moreno PR Muller JE. Identification of high-risk atherosclerotic plaques: a survey of spectroscopic methods. Curr Opin Cardiol. (2002) 17:638–47. 10.1097/00001573-200211000-00010 12466707

[15] Cassis LA Lodder RA. Near-IR imaging of atheromas in living arterial tissue. Anal Chem. (1993) 65:1247–56. 10.1021/ac00057a023 8503505

[16] Dempsey RJ Davis DG Buice RG Lodder RA Biological and medical applications of near-infrared spectrometry. Appl Spectr. (1996) 50:18A–34A. 10.1366/0003702963906537

[17] Moreno PR Lodder RA Purushothaman KR Charash WE O'Connor WN Muller JE. Detection of lipid pool, thin fibrous cap, and inflammatory cells in human aortic atherosclerotic plaques by near-infrared spectroscopy. Circulation. (2002) 105:923–7. 10.1161/hc0802.104291 11864919

[18] Muller JE Abela GS Nesto RW Tofler GH. Triggers, acute risk factors and vulnerable plaques: the lexicon of a new frontier. J Am Coll Cardiol. (1994) 23:809–13. 10.1016/0735-1097(94)90772-2 8113568

[19] Jaross W Neumeister V Lattke P Schuh D. Determination of cholesterol in atherosclerotic plaques using near infrared diffuse reflection spectroscopy. Atherosclerosis. (1999) 147:327–37. 10.1016/S0021-9150(99)00203-8 10559519

[20] Puri R Madder RD Madden SP Sum ST Wolski K Muller JE. Near-infrared spectroscopy enhances intravascular ultrasound assessment of vulnerable coronary plaque: a combined pathological and *in vivo* study. Arterioscler Thromb Vasc Biol. (2015) 35:2423–31. 10.1161/ATVBAHA.115.306118 26338299

[21] Inaba S Mintz GS Burke AP Stone GW Virmani R Matsumura M. Intravascular ultrasound and near-infrared spectroscopic characterization of thin-cap fibroatheroma. Am J Cardiol. (2017) 119:372–8. 10.1016/j.amjcard.2016.10.031 27876264

[22] Abdel-Karim ARR Rangan BV Banerjee S Brilakis ES. Intercatheter reproducibility of near-infrared spectroscopy for the *in vivo* detection of coronary lipid core plaques. Catheter Cardiovasc Interv. (2011) 77:657–61. 10.1002/ccd.22763 20824764

[23] Garcia BA Wood F Cipher D Banerjee S Brilakis ES. Reproducibility of near-infrared spectroscopy for the detection of lipid core coronary plaques and observed changes after coronary stent implantation. Catheter Cardiovasc Interv. (2010) 76:359–65. 10.1002/ccd.22500 20839348

[24] Schultz CJ Serruys PW van der Ent M Ligthart J Mastik F Garg S. First-in-man clinical use of combined near-infrared spectroscopy and intravascular ultrasound: a potential key to predict distal embolization and no-reflow? J Am Coll Cardiol. (2010) 56:314. 10.1016/j.jacc.2009.10.090 20633824

[25] Meese TM Lisauskas JB Doucet C Clark N Gardner CM Hull EL Spectroscopic identification of lipid-rich plaques causing intermediate stenosis: a study in coronary autopsy specimens. Cardiovasc Revasc Med. (2008) 9:108 10.1016/j.carrev.2008.02.026

[26] Grainger SJ Su JL Greiner CA Saybolt MD Wilensky RL Raichlen JS Ability of combined Near-Infrared Spectroscopy-Intravascular Ultrasound (NIRS-IVUS) imaging to detect lipid core plaques and estimate cap thickness in human autopsy coronary arteries. In: Photons Plus Ultrasound: Imaging and Sensing. San Francisco, CA: International Society for Optics and Photonics (2016). 97084V 10.1117/12.2209664

[27] Narula J Nakano M Virmani R Kolodgie FD Petersen R Newcomb R. Histopathologic characteristics of atherosclerotic coronary disease and implications of the findings for the invasive and noninvasive detection of vulnerable plaques. J Am Coll Cardiol. (2013) 61:1041–51. 10.1016/j.jacc.2012.10.054 23473409 PMC3931303

[28] Madder RD Smith JL Dixon SR Goldstein JA. Composition of target lesions by near-infrared spectroscopy in patients with acute coronary syndrome versus stable angina. Circ Cardiovasc Interv. (2012) 5:55–61. 10.1161/CIRCINTERVENTIONS.111.963934 22253357

[29] Madder RD Goldstein JA Madden SP Puri R Wolski K Hendricks M. Detection by near-infrared spectroscopy of large lipid core plaques at culprit sites in patients with acute ST-segment elevation myocardial infarction. JACC Cardiovasc Interv. (2013) 6:838–46. 10.1016/j.jcin.2013.04.012 23871513

[30] Patel D Hamamdzic D Llano R Patel D Cheng L Fenning RS. Subsequent development of fibroatheromas with inflamed fibrous caps can be predicted by intracoronary near infrared spectroscopy. Arterioscler Thromb Vasc Biol. (2013) 33:347–53. 10.1161/ATVBAHA.112.300710 23288155

[31] Schuurman AS Vroegindewey M Kardys I Oemrawsingh RM Cheng JM de Boer S. Near-infrared spectroscopy-derived lipid core burden index predicts adverse cardiovascular outcome in patients with coronary artery disease during long-term follow-up. Eur Heart J. (2018) 39:295–302. 10.1093/eurheartj/ehx247 28531282

[32] Kolodgie FD Burke AP Farb A Gold HK Yuan J Narula J. The thin-cap fibroatheroma: a type of vulnerable plaque: the major precursor lesion to acute coronary syndromes. Curr Opin Cardiol. (2001) 16:285–92. 10.1097/00001573-200109000-00006 11584167

[33] Falk E Shah PK Fuster V. Coronary plaque disruption. Circulation. (1995) 92:657–71. 10.1161/01.CIR.92.3.657 7634481

[34] Karlsson S Anesäter E Fransson K Andell P Persson J Erlinge D. Intracoronary near-infrared spectroscopy and the risk of future cardiovascular events. Open Heart. (2019) 6:e000917. 10.1136/openhrt-2018-000917 30997122 PMC6443121

[35] Goldstein JA Maini B Dixon SR Brilakis ES Grines CL Rizik DG. Detection of lipid-core plaques by intracoronary near-infrared spectroscopy identifies high risk of periprocedural myocardial infarction. Circ Cardiovasc Interv. (2011) 4:429–37. 10.1161/CIRCINTERVENTIONS.111.963264 21972399

[36] Kini AS Baber U Kovacic JC Limaye A Ali ZA Sweeny J. Changes in plaque lipid content after short-term intensive versus standard statin therapy: the YELLOW trial (reduction in yellow plaque by aggressive lipid-lowering therapy). J Am Coll Cardiol. (2013) 62:21–9. 10.1016/j.jacc.2013.03.058 23644090

[37] Oemrawsingh RM Cheng JM García-García HM van Geuns RJ de Boer SPM Simsek C. Near-infrared spectroscopy predicts cardiovascular outcome in patients with coronary artery disease. J Am Coll Cardiol. (2014) 64:2510–8. 10.1016/j.jacc.2014.07.998 25500237

[38] Stone GW Maehara A Muller JE Rizik DG Shunk KA Ben-Yehuda O. Plaque characterization to inform the prediction and prevention of periprocedural myocardial infarction during percutaneous coronary intervention: the CANARY trial (coronary assessment by near-infrared of atherosclerotic rupture-prone yellow). JACC Cardiovasc Interv. (2015) 8:927–36. 10.1016/j.jcin.2015.01.032 26003018

[39] Simsek C Garcia-Garcia HM van Geuns RJ Magro M Girasis C van Mieghem N. The ability of high dose rosuvastatin to improve plaque composition in non-intervened coronary arteries: rationale and design of the integrated biomarker and imaging study-3 (IBIS-3). EuroIntervention. (2012) 8:235–41. 10.4244/EIJV8I2A37 22717926

[40] Oemrawsingh RM Garcia-Garcia HM van Geuns RJM Lenzen MJ Simsek C de Boer SPM. Integrated biomarker and imaging study 3 (IBIS-3) to assess the ability of rosuvastatin to decrease necrotic core in coronary arteries. EuroIntervention. (2016) 12:734–9. 10.4244/EIJV12I6A118 27542785

[41] Kini AS Vengrenyuk Y Shameer K Maehara A Purushothaman M Yoshimura T. Intracoronary imaging, cholesterol efflux, and transcriptomes after intensive statin treatment: the YELLOW II study. J Am Coll Cardiol. (2017) 69:628–40. 10.1016/j.jacc.2016.10.029 27989886

[42] Waksman R Torguson R Spad MA Garcia-Garcia H Ware J Wang R. The lipid-rich plaque study of vulnerable plaques and vulnerable patients: study design and rationale. Am Heart J. (2017) 192:98–104. 10.1016/j.ahj.2017.02.010 28938968

[43] Waksman R Mario CD Torguson R Ali ZA Singh V Skinner WH. Identification of patients and plaques vulnerable to future coronary events with near-infrared spectroscopy intravascular ultrasound imaging: a prospective, cohort study. Lancet. (2019) 394:1629–37. 10.1016/S0140-6736(19)31794-5 31570255

[44] Stone GW Maehara A Lansky AJ de Bruyne B Cristea E Mintz GS. A prospective natural-history study of coronary atherosclerosis. N Engl J Med. (2011) 364:226–35. 10.1056/NEJMoa1002358 21247313

[45] de Boer S Baran Y Garcia-Garcia HM Eskin I Lenzen MJ Kleber ME. The european collaborative project on inflammation and vascular wall remodeling in atherosclerosis - intravascular ultrasound (ATHEROREMO-IVUS) study. EuroIntervention. (2018) 14:194–203. 10.4244/EIJ-D-17-00180 28943493

[46] Calvert PA Obaid DR O'Sullivan M Shapiro LM McNab D Densem CG. Association between IVUS findings and adverse outcomes in patients with coronary artery disease: the VIVA (VH-IVUS in Vulnerable Atherosclerosis) Study. JACC Cardiovasc Imaging. (2011) 4:894–901. 10.1016/j.jcmg.2011.05.005 21835382

[47] Matsumura M Mintz GS Kang SJ Sum ST Madden SP Burke AP. Intravascular ultrasound and near-infrared spectroscopic features of coronary lesions with intraplaque haemorrhage. Eur Heart J Cardiovasc Imaging. (2017) 18:1222–8. 10.1093/ehjci/jew217 28017925

[48] Dixon SR Grines CL Munir A Madder RD Safian RD Hanzel GS. Analysis of target lesion length before coronary artery stenting using angiography and near-infrared spectroscopy versus angiography alone. Am J Cardiol. (2012) 109:60–6. 10.1016/j.amjcard.2011.07.068 21962996

[49] Goldstein JA Grines C Fischell T Virmani R Rizik D Muller J. Coronary embolization following balloon dilation of lipid-core plaques. JACC Cardiovasc Imaging. (2009) 2:1420–4. 10.1016/j.jcmg.2009.10.003 20083078

[50] Raghunathan D Abdel-Karim ARR Papayannis AC daSilva M Jeroudi OM Rangan BV. Relation between the presence and extent of coronary lipid core plaques detected by near-infrared spectroscopy with postpercutaneous coronary intervention myocardial infarction. Am J Cardiol. (2011) 107:1613–8. 10.1016/j.amjcard.2011.01.044 21575750

[51] Saeed B Banerjee S Brilakis ES. Slow flow after stenting of a coronary lesion with a large lipid core plaque detected by near-infrared spectroscopy. EuroIntervention. (2010) 6:545. 10.4244/EIJ30V6I4A90 20884445

[52] Štěchovský C Hájek P Horváth M Špaček M Veselka J. Near-infrared spectroscopy combined with intravascular ultrasound in carotid arteries. Int J Cardiovasc Imaging. (2016) 32:181–8. 10.1007/s10554-015-0687-x 26044524

[53] Horváth M Hájek P Štěchovský C Honěk J Veselka J. Intravascular near-infrared spectroscopy: a possible tool for optimizing the management of carotid artery disease. Int J Angiol. (2015) 24:198–204. 10.1055/s-0035-1558644 26417188 PMC4572008

[54] Abbas AE Zacharias SK Goldstein JA Hanson ID Safian RD. Invasive characterization of atherosclerotic plaque in patients with peripheral arterial disease using near-infrared spectroscopy intravascular ultrasound. Catheter Cardiovasc Interv. (2017) 90:461–70. 10.1002/ccd.27023 28303659

[55] Zacharias SK Safian RD Madder RD Hanson ID Pica MC Smith JL. Invasive evaluation of plaque morphology of symptomatic superficial femoral artery stenoses using combined near-infrared spectroscopy and intravascular ultrasound. Vasc Med. (2016) 21:337–44. 10.1177/1358863X16631420 26957574

[56] Danek BA Karatasakis A Karacsonyi J Alame A Resendes E Kalsaria P. Long-term follow-up after near-infrared spectroscopy coronary imaging: insights from the lipid cORe plaque association with clinical events (ORACLE-NIRS) registry. Cardiovasc Revasc Med. (2017) 18:177–81. 10.1016/j.carrev.2016.12.006 28017258

[57] Madder RD Husaini M Davis AT VanOosterhout S Khan M Wohns D. Large lipid-rich coronary plaques detected by near-infrared spectroscopy at non-stented sites in the target artery identify patients likely to experience future major adverse cardiovascular events. Eur Heart J Cardiovasc Imaging. (2016) 17:393–9. 10.1093/ehjci/jev340 26800770

[58] Dohi T Maehara A Moreno PR Baber U Kovacic JC Limaye AM. The relationship among extent of lipid-rich plaque, lesion characteristics, and plaque progression/regression in patients with coronary artery disease: a serial near-infrared spectroscopy and intravascular ultrasound study. Eur Heart J Cardiovasc Imaging. (2015) 16:81–7. 10.1093/ehjci/jeu169 25190072 PMC4357809

[59] Fard AM Vacas-Jacques P Hamidi E Wang H Carruth RW Gardecki JA. Optical coherence tomography–near infrared spectroscopy system and catheter for intravascular imaging. Opt Express. (2013) 21:30849–58. 10.1364/OE.21.030849 24514658 PMC3926541

